# Editorial: Current and Future Developments in the Therapeutic Management of Neuromuscular Diseases

**DOI:** 10.3389/fneur.2021.835839

**Published:** 2022-01-17

**Authors:** George K. Papadimas, Roser Pons, Johanna Palmio

**Affiliations:** ^1^1st Department of Neurology, Eginitio Hospital, National and Kapodistrian University of Athens, Athens, Greece; ^2^1st Department of Pediatrics, Aghia Sofia Hospital, National and Kapodistrian University of Athens, Athens, Greece; ^3^Department of Neurology, Neuromuscular Research Center, Tampere University Hospital and Tampere University, Tampere, Finland

**Keywords:** neuromuscular diseases, myopathy, neuropathy, myasthenia, treatment

Neuromuscular diseases cover a wide range of acquired and inherited neuropathies, myopathies and junction disorders. For most of them, treatment options were, until recently, extremely poor or even non-existent and often recommendations have been limited to conservative measures, physical activity and life style modifications.

In recent years, the development and application of new more effective diagnostic tools, such as modern imaging techniques, histopathological studies and advanced genetics and a better in-depth understanding of their underlying pathomechanisms, have led to an earlier diagnosis and improved therapeutic management (1). In addition, the availability of several alternative treatments requires more sophisticated selection of patients based on various criteria.

Myasthenia Gravis (MG) is the most common neuromuscular transmission disorder (2). Despite the existence of few refractory cases, the goal of treatment is the complete remission of symptoms and the selection of the therapeutic strategy should rely on phenotypical characteristics, serological subtypes and patients' comorbidities (3). In the research paper by Moodley et al. the authors investigated the different clinical characteristics and outcomes in HIV-infected and HIV-uninfected MG patients and concluded that the former were more refractory to immunosuppressive treatments and were probably more likely to require combination rescue therapy with PE/IVIG and IV cyclophosphamide compared to HIV-uninfected MG patients. A systematic review and meta-analysis by Feng et al. focused specifically on the optimal treatments for refractory MG and according to the results, rituximab and eculizumab proved to be both effective, although rituximab seemed to be safer with fewer adverse events. In a brief research report by Guan et al. the authors identified two novel variants in *HADHB* gene, in a patient with Charcot-Marie-Tooth disease (CMT) and rabdomyolysis and expanded accordingly the clinical spectrum of these disorders. Notably, the patient responded satisfactorily to the appropriate dietary modifications and the restriction of long-chain fatty acid. Immune-mediated necrotizing myopathy (IMNM) is one of the commonest forms of inflammatory myopathies, associated in most cases, with anti-3-hydroxy-3-methylglutaryl-coA reductase (anti-HMGCR) myositis-specific autoantibodies or anti-signal recognition particle (anti-SRP), although about 20% of patients are double seronegative (4). In a research paper by Ma et al. the authors compared the clinical and pathological features between seropositive and seronegative patients with IMNM and found that the latter presented more frequently with myalgia, exhibited more subclinical cardiac involvement, tended to respond better to immunotherapy and showed a better outcome. Finally, the research paper by Marchetti et al. focused on the investigation of possible determinants of disease progression in patients with LGMDR4. The age of disease onset is a well-known independent predictor of severity in patients with sarcoglycanopathies (5). This study showed that CK decrease reflects the worsening of muscle function and can also predict clinical outcomes, such as the need for respiratory assistance, while EF (ejection fraction) was the strongest independent variable for the progression of the disease. The above data may be useful for understanding the disease natural history and may be also taken into consideration for further clinical trials.

## Author Contributions

GP served as a topic editor. RP and JP as topic co-editors for the Research Topic Current and Future Developments in the Therapeutic Management of Neuromuscular Diseases. All authors contributed to the article and approved the submitted version.

## Conflict of Interest

The authors declare that the research was conducted in the absence of any commercial or financial relationships that could be construed as a potential conflict of interest.

## Publisher's Note

All claims expressed in this article are solely those of the authors and do not necessarily represent those of their affiliated organizations, or those of the publisher, the editors and the reviewers. Any product that may be evaluated in this article, or claim that may be made by its manufacturer, is not guaranteed or endorsed by the publisher.
